# Shock Timing in STEMI-CS: Management and Outcomes in a Resource-Limited Setting

**DOI:** 10.1016/j.jacadv.2026.103179

**Published:** 2026-09-23

**Authors:** Luis Enrique Remedios Carbonell, Mariaydis Perez Reyes, Dayani Arteaga Guerra, Maidelis Prieto Guerra, Geovedy Martinez Garcia, Maikel Santos Medina, Leonardo Almaguer Gongora, Bernardo Reyes, Miguel Rodriguez Ramos

**Affiliations:** a Internal Medicine Resident, HCA Florida Aventura Hospital, Aventura, Florida, USA; b Lifetime Medical Associates, Aventura, Florida, USA; c Emergency Medical Department, Hospital Universitario de Canarias, Santa Cruz de Tenerife, Spain; d Department of Cardiology, Hospital Hermanos Ameijeiras, Havana, Cuba; e Cardiocentro Uruapan, Uruapan, Michoacan, Mexico; f Department of Cardiology, Hospital General Ernesto Guevara, Las Tunas, Cuba; g Internal Medicine Residency Program Director, HCA Florida Aventura Hospital, Aventura, Florida, USA; h Division of Internal Medicine, Dr. Kiran C. Patel College of Allopathic Medicine (NSU MD), NOVA Southeastern University, Davie, Florida, USA

**Keywords:** cardiogenic shock, mortality, reperfusion, resource-limited setting, STEMI

## Abstract

**Background:**

Cardiogenic shock complicating ST-segment elevation myocardial infarction (STEMI) carries high in-hospital mortality, but evidence from low- and middle-income countries remains limited.

**Objectives:**

The objective of the study was to compare clinical presentation, pharmacological treatment use, and in-hospital outcomes according to shock onset before vs after arrival at a reperfusion center (ARC).

**Methods:**

We performed a retrospective cohort analysis with data from RECUIMA, a multicenter registry of patients with STEMI admitted to 4 Cuban hospitals. Patients with cardiogenic shock complicating STEMI were classified as having shock prior to arrival at a reperfusion center (PRC) or ARC. Multivariable logistic regression assessed the association between shock timing and in-hospital mortality.

**Results:**

Among 4,113 patients with STEMI, 435 (10.6%) developed cardiogenic shock; 134 (30.8%) were PRC and 301 (69.2%) ARC. PRC patients had greater clinical severity, including lower systolic blood pressure and left ventricular ejection fraction, worse renal function, and higher Global Registry of Acute Coronary Events scores (all *P* < 0.01). Inpatient best available medical therapy was less frequent in PRC than ARC patients (32.8% vs 51.8%; relative risk: 0.63; 95% CI: 0.49-0.83; *P* < 0.001). Unadjusted mortality was higher in PRC patients (50.0% vs 38.2%; relative risk: 1.31; 95% CI: 1.05-1.63; *P* = 0.021). In the multivariable logistic regression model, shock timing was not statistically significantly associated with mortality (OR: 1.65; 95% CI: 0.86-3.18; *P* = 0.13).

**Conclusions:**

PRC patients presented with greater severity, received less inpatient best available medical therapy, and had higher unadjusted mortality. However, in the multivariable logistic regression model, shock timing was not statistically significantly associated with mortality.

Cardiogenic shock complicating ST-segment elevation myocardial infarction (STEMI) (STEMI-CS) is associated with high mortality, impaired quality of life among survivors, and substantial health care resource utilization. Despite advances in reperfusion strategies, mechanical circulatory support, and critical care infrastructure, contemporary registries from high-income countries continue to report persistently high mortality rates.^1^ In contrast, evidence from low- and middle-income countries remains limited, underscoring the need for broader and more globally representative data.^2^^,^^3^

In resource-limited settings, delayed arrival to hospital after symptom onset may reduce the opportunity for timely and effective reperfusion. In addition, limited access to cardiac catheterization laboratories, intensive care units with advanced hemodynamic monitoring, and personnel trained to manage complex cardiovascular emergencies may further compromise care delivery.^4^ These systemic challenges, together with the growing burden of cardiovascular disease in developing regions, may contribute to disproportionately poor outcomes.^5^^,^^6^

RECUIMA (Registro Cubano de Infarto Agudo de Miocardio) is a prospective, multicenter observational registry established to systematically collect standardized clinical data on consecutive patients with STEMI admitted to 4 participating hospitals in Cuba. The registry captures demographic, clinical, laboratory, pharmacological treatment, and outcome data using harmonized data collection protocols to evaluate real-world management strategies and clinical outcomes in routine practice.

Using data from RECUIMA, we sought to compare clinical presentation, pharmacological treatment use, and in-hospital outcomes between patients who developed STEMI-CS prior to arrival at a reperfusion center (PRC) and those who developed shock after arrival in a resource-limited setting.

The study design, patient classification, principal findings, and clinical implications are summarized in the Central Illustration.

**Central Illustration:**
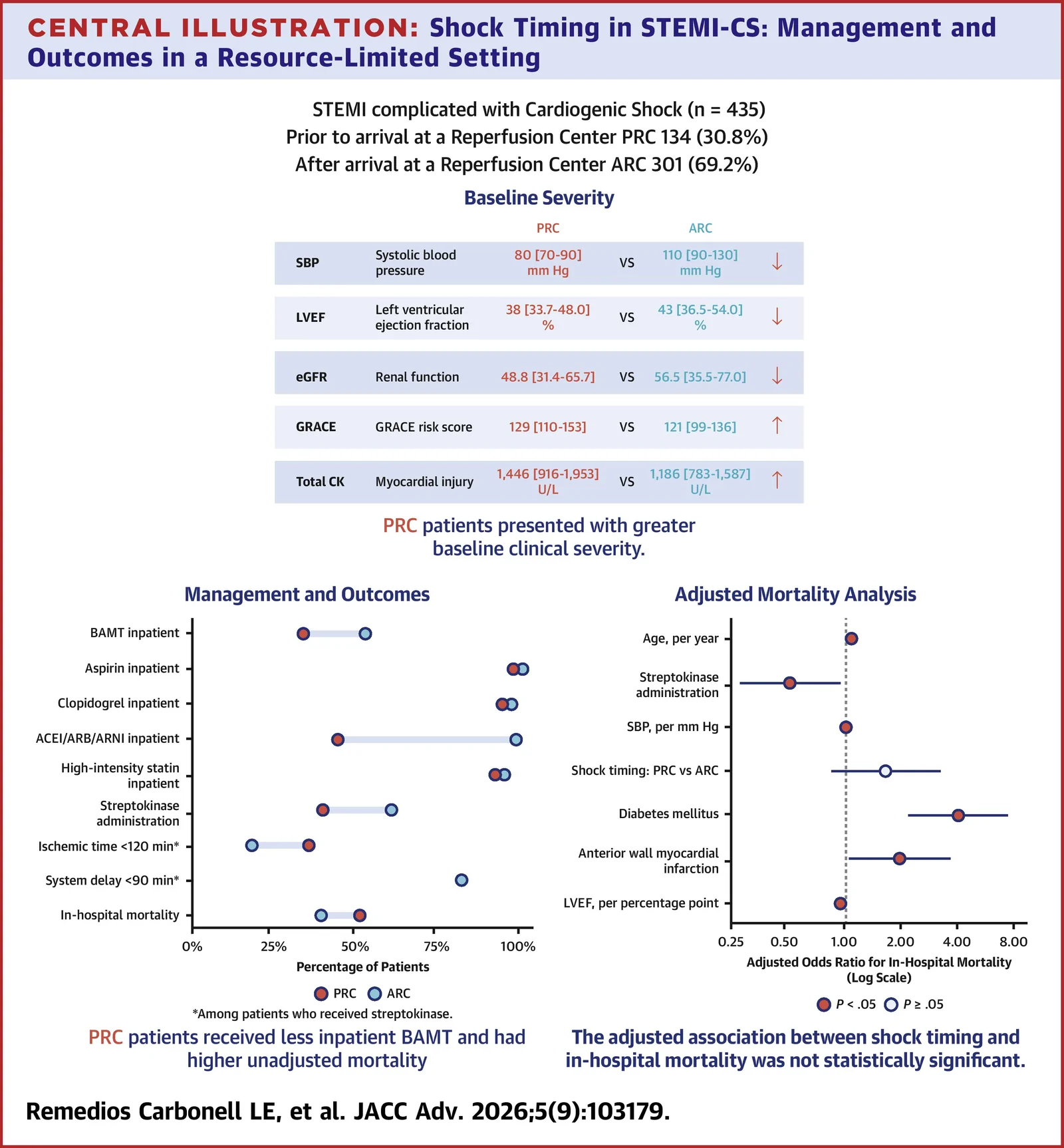
Shock Timing in STEMI-CS: Management and Outcomes in a Resource-Limited Setting This multicenter registry evaluated patients with ST-segment elevation myocardial infarction complicated by cardiogenic shock (STEMI-CS) in a resource-limited setting. Patients were classified according to shock onset prior to arrival at a reperfusion center (PRC) or after arrival at a reperfusion center (ARC). PRC patients presented with greater baseline clinical severity, received less inpatient BAMT and streptokinase, and had higher unadjusted in-hospital mortality. In the multivariable logistic regression model, the association between PRC status and in-hospital mortality did not reach statistical significance (OR: 1.65; 95% CI: 0.86-3.18; *P* = 0.13). ACE = angiotensin-converting enzyme; ARB = angiotensin II receptor blocker; ARNI = angiotensin receptor-neprilysin inhibitor; BAMT = best available medical therapy; CK = creatine kinase; eGFR = estimated glomerular filtration rate; GRACE = Global Registry of Acute Coronary Events; LVEF = left ventricular ejection fraction; STEMI = ST-segment elevation myocardial infarction.

## Methods

### Study design and population

This observational cohort study included consecutive adult patients with STEMI-CS from RECUIMA, a multicenter observational registry enrolling adult patients admitted to 3 regional secondary-level hospitals and 1 tertiary care center within the Cuban health system. The registry enrolled patients between 2019 and 2024 and collected clinical and therapeutic data from patients with acute myocardial infarction, with the aim of characterizing STEMI in the country and optimizing cardiovascular care.^7^

No exclusion criteria were applied. Baseline data, care and process metrics, adverse events, and in-hospital outcomes were collected and retrospectively adjudicated according to published standards. Patients and the public were not involved in the design, conduct, reporting, or dissemination plans of this research.

### Variable definition

STEMI-CS was defined as STEMI resulting in clinical and biochemical evidence of sustained tissue hypoperfusion, characterized by systolic blood pressure <90 mm Hg for ≥30 minutes, or the need for vasopressors, inotropes, or mechanical circulatory support to maintain systolic blood pressure ≥90 mm Hg. This was accompanied by evidence of end-organ hypoperfusion, such as cold extremities, altered mental status, or oliguria, and evidence of myocardial dysfunction confirmed by clinical, laboratory, or imaging findings, as suggested by the Shock Academic Research Consortium Scientific Expert Panel.^8^

Among patients presenting with STEMI, 2 groups were defined: patients who developed cardiogenic shock PRC and patients who developed cardiogenic shock after arrival at a reperfusion center (ARC). Admission systolic blood pressure was defined as the first documented systolic blood pressure on arrival at the participating hospital. Ischemic time was defined as the interval from the onset of chest pain or ischemic-equivalent symptoms to fibrinolytic administration, corresponding to symptom-to-needle time. System delay was defined as the time from the first medical contact to the initiation of streptokinase administration.

Best available medical therapy (BAMT), used as a surrogate for guideline-directed medical therapy given the resource-limited setting of the registry, was defined as the best evidence-based therapy available in the Cuban medication formulary. Inpatient BAMT was defined as the combined prescription of aspirin, P2Y_12_ inhibitors, statins, angiotensin-converting enzyme inhibitors/angiotensin receptor blockers/angiotensin receptor-neprilysin inhibitors, and fibrinolytic therapy according to national protocols. BAMT at discharge was defined as the combined prescription of aspirin, P2Y_12_ inhibitors, angiotensin-converting enzyme inhibitors/angiotensin receptor blockers/angiotensin receptor-neprilysin inhibitors, beta-blockers, and statins. Administration of angiotensin-converting enzyme inhibitors/angiotensin receptor blockers/angiotensin receptor-neprilysin inhibitors and beta-blockers was defined as BAMT for reduced left ventricular ejection fraction (LVEF) (≤40%).

### Statistical analysis

Continuous variables are presented as median and 25th–75th percentiles (Q1–Q3) and were compared between the PRC and ARC groups using the Mann-Whitney *U* test. Categorical variables are presented as n (%) in Table 1 and as frequencies in Table 2, Table 3, Table 4, unless otherwise indicated, and were compared using Pearson’s chi-square test or Fisher exact test, as appropriate. Associations between PRC vs ARC status and categorical treatment, process, and outcome variables were expressed as relative risks (RRs) with 95% CIs calculated using the log-Wald method. All statistical tests were 2-sided, and a *P* value <0.05 was considered statistically significant. IBM SPSS Statistics (version 24) was used for analysis.

**Table 1:** Characteristics and Clinical Profile of Patients With STEMI-CS According to Shock Onset Timing

	PRC (n = 134)	ARC (n = 301)	*P* Value
Age, y	71 [61.5–80]	70 [59–77]	0.27
Female	62 (46.3)	120 (39.9)	0.21
Male	72 (53.7)	181 (60.1)	
Diabetes mellitus type 2	51 (38.1)	98 (32.6)	0.26
Hypertension	108 (80.6)	239 (79.4)	0.78
Known coronary artery disease	40 (29.9)	79 (26.2)	0.44
Prior myocardial infarction	13 (9.7)	28 (9.3)	0.90
Prior stroke	5 (3.7)	18 (6.0)	0.33
Peripheral artery disease	11 (8.2)	12 (4.0)	0.069
COPD	7 (5.2)	7 (2.3)	0.14
Dyslipidemia	9 (6.7)	24 (8.0)	0.65
Atrial fibrillation	5 (3.7)	5 (1.7)	0.30
Congestive heart failure	4 (3.0)	9 (3.0)	1.00
Current smoker	70 (52.2)	154 (51.2)	0.84
Left ventricular ejection fraction, %	38 [33.7–48.0]	43 [36.5–54.0]	<0.001
eGFR, mL/min/1.73 m^2^	48.8 [31.4–65.7]	56.5 [35.5–77.0]	<0.001
Glycemia, mmol/L	7.8 [6.3–11.1]	6.8 [5.7–9.3]	0.078
Cholesterol, mmol/L	4.7 [4.2–5.4]	4.7 [3.8–5.5]	0.94
Total CK, U/L	1,446 [916–1,953]	1,186 [783–1,587]	0.009
Systolic blood pressure at admission, mm Hg	80 [70–90]	110 [90–130]	<0.001
Triglycerides, mmol/L	1.3 [1.0–1.9]	1.2 [0.9–1.6]	0.046
GRACE score at admission	129 [110–153]	121 [99–136]	<0.001
Length of stay among survivors, days	8 [7–9]	9 [7–11]	0.021
Length of stay among nonsurvivors, days	4 [1–5]	4 [2–5]	0.89

Values are median [15th-75th percentile, Q1-Q3] or n (%).

ARC = after arrival at a reperfusion center; CK = creatine kinase; COPD = chronic obstructive pulmonary disease; eGFR = estimated glomerular filtration rate; GRACE = Global Registry of Acute Coronary Events; PRC = prior to arrival at a reperfusion center; STEMI-CS = ST-segment elevation myocardial infarction with cardiogenic shock.

**Table 2:** In-Hospital Quality-of-Care Measures and Outcomes in Patients With STEMI-CS

	PRC (n = 134)	ARC (n = 301)	Relative Risk	95% CI	*P* Value
BAMT inpatient	44	156	0.63	0.49–0.83	<0.001
Aspirin inpatient	130	298	0.98	0.95–1.01	0.21
Clopidogrel inpatient	125	288	0.97	0.93–1.03	0.29
ACEI/ARB/ARNI inpatient	58	293	0.44	0.37–0.54	<0.001
High-intensity statin inpatient	122	282	0.97	0.91–1.03	0.32
Streptokinase administration	52	179	0.65	0.52–0.82	<0.001
Ischemic time <120 min	18/52	31/179	2.00	1.22–3.27	0.007
System delay time <90 min	42/52	145/179	1.00	0.86–1.16	0.97
In-hospital mortality	67	115	1.31	1.05–1.63	0.021

ACEI = angiotensin-converting enzyme inhibitor; ARB = angiotensin II receptor blocker; ARNI = angiotensin receptor-neprilysin inhibitor; BAMT = best available medical therapy; other abbreviations as in Table 1.

Values are n unless otherwise indicated. RRs and 95% CIs were calculated using the log-Wald method. *P* values were derived from Pearson’s chi-square test or Fisher exact test, as appropriate.

**Table 3:** Medical Therapy Among Patients With Reduced LVEF Discharged Alive After STEMI-CS

	PRC (n = 30)	ARC (n = 49)	Relative Risk	95% CI	*P* Value
BAMT	20	18	1.81	1.16–2.84	0.010
Beta-blockers	23	27	1.39	1.01–1.92	0.054
ACEI/ARB/ARNI	24	37	1.06	0.83–1.35	0.64

LVEF = left ventricular ejection fraction; other abbreviations as in Tables 1 and 2.

Values are n unless otherwise indicated. RRs and 95% CIs were calculated using the log-Wald method. *P* values were derived from Pearson’s chi-square test or Fisher’s exact test, as appropriate.

**Table 4:** Discharge Medical Therapy Among Survivors of STEMI-CS

	PRC (n = 67)	ARC (n = 186)	Relative Risk	95% CI	*P* Value
BAMT at discharge	34	62	1.52	1.12–2.08	0.012
Aspirin at discharge	65	177	1.02	0.97–1.08	0.73
Clopidogrel at discharge	64	177	1.00	0.94–1.07	1.00
Beta-blockers	39	80	1.35	1.04–1.76	0.033
ACEI/ARB/ARNI at discharge	49	134	1.02	0.86–1.20	0.86
High-intensity statin at discharge	64	176	1.01	0.95–1.07	1.00

Abbreviations as in Tables 1 and 2.

Values are n unless otherwise indicated. RRs and 95% CIs were calculated using the log-Wald method. *P* values were derived from Pearson’s chi-square test or Fisher exact test, as appropriate.

Missing data accounted for <5% of all variables. Given the low frequency of missingness, missing values were addressed using single imputation. Missing continuous values were imputed using the overall cohort mean for approximately normally distributed variables and the overall cohort median for skewed variables. Missing categorical values were imputed using the modal category. Multicollinearity among covariates was assessed using variance inflation factor analysis before model construction, and variables with variance inflation factor >5 were evaluated before final inclusion in the model. Univariable and multivariable logistic regression models were used to evaluate the association of selected variables with in-hospital mortality. The multivariable model included age, streptokinase administration, admission systolic blood pressure, shock timing, diabetes mellitus, anterior wall myocardial infarction, and LVEF. All covariates were entered simultaneously using the enter method. Variables were selected according to clinical relevance and previously established associations with mortality in STEMI-CS. Logistic regression model results are presented as the OR with 95% CI. Forest and dumbbell plots were generated using R statistical software (v4.5.2; R Core Team 2025).

The Ethics Committee of Camilo Cienfuegos General Hospital approved the exemption from informed consent given the retrospective design of the study and the use of previously collected clinical data.

## Results

Among 4,113 patients included in the RECUIMA database, 435 met the clinical or hemodynamic criteria for cardiogenic shock at some point during their clinical course. Of these, 134 developed cardiogenic shock PRC, whereas 301 developed cardiogenic shock ARC.

### Patient characteristics

Baseline demographic characteristics, selected comorbidities, and other clinical variables of the study population are presented in Table 1. In the PRC group, the median age was 71 years (Q1–Q3: 61.5-80), compared with 70 years (Q1–Q3: 59-77) in the ARC group. Women represented 46.3% (n = 62) of the PRC group and 39.9% (n = 120) of the ARC group. Hypertension was the most prevalent comorbidity in both cohorts, affecting 80.6% (n = 108) of patients in the PRC group and 79.4% (n = 239) in the ARC group. No statistically significant differences were observed between groups with respect to age (*P* = 0.27) or sex (*P* = 0.21).

LVEF was lower in the PRC group than in the ARC group (median: 38% [Q1–Q3: 33.7-48.0] vs 43% [Q1–Q3: 36.5-54.0]; *P* < 0.001). Similarly, estimated glomerular filtration rate was lower in the PRC group (median: 48.8 mL/min/1.73 m^2^ [Q1–Q3: 31.4-65.7]) than in the ARC group (56.5 mL/min/1.73 m^2^ [Q1–Q3: 35.5-77.0]; *P* < 0.001). Systolic blood pressure at admission was also lower in the PRC group (median: 80 mm Hg [Q1–Q3: 70-90] vs 110 mm Hg [Q1–Q3: 90-130]; *P* < 0.001). Total creatine kinase levels were higher in PRC patients than in ARC patients (median: 1,446 U/L [Q1–Q3: 916-1,953] vs 1,186 U/L [Q1–Q3: 783-1,587]; *P* = 0.009). The Global Registry of Acute Coronary Events score at admission was also higher in the PRC group (median: 129 [Q1–Q3: 110-153] vs 121 [Q1–Q3: 99-136]; *P* < 0.001).

### Management and clinical outcomes

Quality-of-care measures in patients with STEMI-CS are shown in Table 2. Inpatient BAMT differed significantly between cohorts and was used less frequently in the PRC group than in the ARC group (32.8% vs 51.8%; RR: 0.63; 95% CI: 0.49-0.83; *P* < 0.001), representing a 37% relative reduction in the likelihood of receiving BAMT and an absolute difference of 19 percentage points.

Furthermore, patients in the PRC group were 35% less likely to receive streptokinase than those in the ARC group (38.8% vs 59.5%; RR: 0.65; 95% CI: 0.52-0.82; *P* < 0.001), corresponding to an absolute difference of 20.7 percentage points. Among patients who received streptokinase, those in the PRC group were twice as likely to achieve an ischemic time <120 minutes compared with those in the ARC group (34.6% vs 17.3%; RR: 2.00; 95% CI: 1.22-3.27; *P* = 0.007).

In-hospital mortality was significantly higher in the PRC group than in the ARC group (50.0% vs 38.2%; RR: 1.31; 95% CI: 1.05-1.63; *P* = 0.021), indicating a 31% relative increase in risk and an absolute risk difference of 11.8%.

Among patients discharged alive with reduced LVEF (Table 3), those in the PRC group had an 81% higher likelihood of receiving BAMT compared with those in the ARC group (66.7% vs 36.7%; RR: 1.81; 95% CI: 1.16-2.84; *P* = 0.010). Beta-blocker use was numerically higher in the PRC group but did not meet the prespecified threshold for statistical significance (76.7% vs 55.1%; RR: 1.39; 95% CI: 1.01-1.92; *P* = 0.054). No significant difference was observed in angiotensin-converting enzyme (ACE) inhibitor/angiotensin II receptor blocker (ARB)/angiotensin receptor-neprilysin inhibitor (ARNI) use (80.0% vs 75.5%; RR: 1.06; 95% CI: 0.83-1.35; *P* = 0.64).

As shown in Table 1, among patients discharged alive, the median length of stay was shorter in the PRC group than in the ARC group (8 days [Q1-Q3: 7-9] vs 9 days [Q1-Q3: 7-11]; *P* = 0.021). BAMT at discharge was more frequently prescribed in the PRC group than in the ARC group (50.7% vs 33.3%; RR: 1.52; 95% CI: 1.12-2.08; *P* = 0.012).

Among survivors, beta-blocker prescription rates at discharge were significantly higher in the PRC group than in the ARC group (58.2% vs 43.0%; RR: 1.35; 95% CI: 1.04-1.76; *P* = 0.033) (Table 4).

No statistically significant differences in discharge prescription rates were observed for aspirin (*P* = 0.73), clopidogrel (*P* = 1.00), high-intensity statins (*P* = 1.00), or ACE inhibitors/ARBs/ARNIs (*P* = 0.86).

In univariable analysis (Table 5), older age, absence of streptokinase administration, PRC status, diabetes mellitus, anterior wall myocardial infarction, and lower LVEF were significantly associated with in-hospital mortality. In the multivariable model, older age (OR: 1.08 per year; 95% CI: 1.05-1.11; *P* < 0.001), diabetes mellitus (OR: 3.99; 95% CI: 2.21-7.22; *P* < 0.001), anterior wall myocardial infarction (OR: 1.95; 95% CI: 1.06-3.59; *P* = 0.032), and higher admission systolic blood pressure (OR: 1.014 per mm Hg; 95% CI: 1.004-1.024; *P* = 0.008) were associated with greater odds of in-hospital mortality. Streptokinase administration (OR: 0.51; 95% CI: 0.28-0.93; *P* = 0.028) and higher LVEF (OR: 0.951 per percentage point; 95% CI: 0.921-0.982; *P* = 0.002) were associated with lower odds of mortality. PRC status was not statistically significantly associated with mortality (OR: 1.65; 95% CI: 0.86-3.18; *P* = 0.13).

**Table 5:** Univariable and Multivariable Logistic Regression for In-Hospital Mortality

	Univariable OR (95% CI)	*P* Value	Multivariable OR (95% CI)	*P* Value
Age, y	1.08 (1.06-1.10)	<0.001	1.08 (1.05-1.11)	<0.001
Streptokinase administration	0.35 (0.24-0.53)	<0.001	0.51 (0.28-0.93)	0.028
Systolic blood pressure at admission, mm Hg	1.01 (0.99-1.02)	0.11	1.014 (1.004-1.024)	0.008
Shock timing (PRC vs ARC)	1.62 (1.07-2.44)	0.022	1.65 (0.86-3.18)	0.13
Diabetes mellitus	2.82 (1.88-4.24)	<0.001	3.99 (2.21-7.22)	<0.001
Anterior wall myocardial infarction	2.16 (1.46-3.19)	<0.001	1.95 (1.06-3.59)	0.032
Left ventricular ejection fraction, %	0.92 (0.89-0.94)	<0.001	0.951 (0.921-0.982)	0.002

Abbreviations as in Table 1.

ARC was the reference category for shock timing. Univariable and multivariable binary logistic regression models were used to identify factors associated with in-hospital mortality. Covariates in the multivariable model were entered simultaneously using the enter method. ORs for age, systolic blood pressure, and left ventricular ejection fraction represent the change in odds associated with a 1-U increase.

## Discussion

### Synthesis of principal findings

In this multicenter cohort study conducted in a resource-limited setting, we evaluated clinical presentation, pharmacological treatment use, and in-hospital outcomes among patients with STEMI-CS. The main finding was that patients who developed cardiogenic shock PRC had higher unadjusted in-hospital mortality than those who developed shock ARC. However, the association between shock timing and mortality was not statistically significant in the multivariable logistic regression model.

A poorer prognosis among patients presenting with cardiogenic shock at admission compared with those who developed shock during hospitalization was reported by Qiao et al^9^ in a cohort study of 675 STEMI patients undergoing primary percutaneous coronary intervention (pPCI). However, that study included only patients treated with pPCI, which limits direct comparison with our cohort. In contrast, another study reported higher mortality and major adverse cardiovascular events rates among patients who developed cardiogenic shock during hospitalization compared with those presenting with shock on arrival.^10^ That study was conducted in a developed-country setting with higher pPCI rates and broader access to guideline-directed medical therapy, which may partly explain differences in outcomes compared with our cohort. In addition, because the reported mortality comparisons were not derived from a multivariable regression model, the independent association between shock timing and mortality remains uncertain.

Overall, the PRC group presented with greater clinical severity at admission, reflected by lower median LVEF, worse renal function, lower blood pressure, higher total creatine kinase levels, and a higher Global Registry of Acute Coronary Events risk score. These findings suggest that shock onset before arrival at a reperfusion center may serve as a marker of more advanced physiological compromise. Although PRC status was associated with mortality in univariable analysis, the association was no longer statistically significant in the multivariable logistic regression model, and the adjusted estimate remained imprecise.

A notable finding was the difference in acute pharmacotherapy between groups. Streptokinase and ACE inhibitor/ARB/ARNI administration during hospitalization were significantly less frequent in the PRC group. These findings are consistent with prior registry data showing that adherence to key management strategies in acute myocardial infarction–related cardiogenic shock may vary substantially and are associated with clinical outcomes.^11^ The lower use of ACE inhibitors, ARBs, or ARNIs in this cohort may be explained by greater hemodynamic instability, as these agents are generally limited in the setting of hypotension or shock. No significant between-group differences were observed in aspirin, clopidogrel, or high-intensity statin administration.

The disparity in streptokinase administration between the PRC and ARC groups likely reflects a complex interaction between greater clinical severity at presentation and systemic limitations inherent to resource-limited settings. Patients in the PRC group presented with more advanced hemodynamic compromise, which may have influenced eligibility for fibrinolytic therapy and shifted the initial clinical focus toward stabilization before reperfusion decisions. In addition, delayed arrival to the reperfusion center may have narrowed the effective therapeutic window and reduced the likelihood of receiving streptokinase.

Importantly, among PRC patients who did receive streptokinase, the likelihood of achieving an ischemic time <120 minutes was twice that observed in the ARC group. This finding suggests that, when PRC patients were considered appropriate candidates for fibrinolysis, treatment was delivered with substantial attention to timeliness goals. Therefore, the lower overall rate of fibrinolysis in the PRC group may be better understood as the result of clinical selection, hemodynamic instability, and delayed presentation rather than a generalized lack of therapeutic effort.

In resource-limited settings, the underuse of fibrinolytic therapy among patients with STEMI is often multifactorial. Delayed presentation remains a major barrier and may reflect limited emergency medical services, long travel distances, inadequate transportation infrastructure, and low public awareness of myocardial infarction symptoms.^12^ Diagnostic barriers may also contribute, as timely reperfusion requires prompt electrocardiographic confirmation of STEMI and the availability of clinicians or trained personnel capable of interpreting the findings. In settings where electrocardiogram availability, interpretation, transmission, and coordination between peripheral facilities and reperfusion-capable centers are limited, diagnosis and treatment decisions may be delayed.

Among patients with reduced LVEF who survived to hospital discharge, BAMT was more frequently prescribed in the PRC group than in the ARC group. Similarly, among patients discharged alive, both overall BAMT and beta-blocker prescription rates were higher in the PRC group. These findings may reflect targeted efforts to optimize postevent management among high-risk survivors once hemodynamic stability has been achieved. However, discharge-treatment comparisons are inherently conditioned on survival and should be interpreted cautiously because of potential survivor bias.

In the multivariable logistic regression model, older age, diabetes mellitus, anterior wall myocardial infarction, higher admission systolic blood pressure, lower LVEF, and absence of streptokinase administration were associated with in-hospital mortality. The associations of age, diabetes, anterior infarction, and reduced LVEF are clinically plausible and consistent with greater comorbidity burden, larger myocardial injury, and impaired cardiac reserve. These findings are broadly consistent with previously reported prognostic factors in cardiogenic shock.^13^ Streptokinase administration was associated with lower mortality; however, this observational association should not be interpreted causally because treatment selection was likely influenced by presentation timing, contraindications, hemodynamic instability, and survival long enough to receive reperfusion therapy.

The positive adjusted association between admission systolic blood pressure and mortality should be interpreted cautiously. This analysis was restricted to patients who developed cardiogenic shock at some point during their clinical course. In PRC patients, admission blood pressure was frequently measured after shock had already developed, whereas ARC patients could have relatively preserved blood pressure at presentation before subsequent hemodynamic deterioration. Admission systolic blood pressure may therefore reflect the temporal stage at which shock was recognized rather than the overall burden or duration of hypotension. Moreover, adjustment for shock timing and other interrelated markers of hemodynamic severity may have altered the conditional association of admission blood pressure with mortality. Accordingly, the positive coefficient should not be interpreted as evidence that higher blood pressure is biologically harmful.

Taken together, these findings highlight the clinical complexity of STEMI-CS in resource-limited settings. Patients presenting with shock before arrival at a reperfusion center represent a particularly high-risk subgroup, characterized by greater baseline severity, lower use of selected acute therapies, and higher unadjusted mortality. However, the observed mortality difference may reflect multiple inter-related clinical, temporal, and treatment-related factors rather than shock timing alone. These results underscore the importance of improving early recognition, reducing delays to presentation, and optimizing reperfusion pathways for patients with STEMI-CS in settings where pPCI is not universally available.^14^, ^15^, ^16^

### Study strengths and limitations

A major strength of this study is its use of data from a multicenter registry in a low- and middle-income country, providing evidence from a resource-limited setting that remains under-represented in cardiovascular research.

Several limitations should also be acknowledged. First, the observational design limits the ability to establish causal relationships between management strategies and clinical outcomes. Second, because participating regional hospitals had limited access to cardiac catheterization laboratories, the findings primarily reflect a reperfusion strategy based on fibrinolytic therapy and may not be fully generalizable to high-income settings where pPCI is the standard of care.

Although key adherence metrics were analyzed, unmeasured confounding may have influenced clinical decision-making regarding BAMT administration, particularly among patients with reduced LVEF. In addition, limited availability of therapies such as ARNIs and sodium glucose co-transporter 2 inhibitors within the Cuban health system formulary, together with restricted access to pPCI, should be considered when interpreting treatment use. Therefore, BAMT was used as a pragmatic surrogate for guideline-directed medical therapy in this resource-limited context.

The higher rate of beta-blocker prescription among PRC survivors at discharge should also be interpreted with caution because of potential survivor bias. Given the higher unadjusted in-hospital mortality observed in the PRC group, patients who survived to discharge likely represented a more clinically stable subgroup capable of tolerating beta-blocker therapy. Thus, this finding may reflect selective survival among patients with prearrival shock rather than a generalized treatment pattern across the entire PRC cohort.

In addition, symptom-to-door time was not consistently recorded across participating centers and therefore could not be included in the analysis. As this variable may influence both the timing of shock recognition and treatment initiation, residual confounding related to delayed presentation cannot be excluded.

Similarly, detailed data on vasoactive therapy, including specific agents, dose, duration, and timing of initiation, were outside the intended scope of the registry and were not systematically recorded. These treatment details may have provided additional insight into shock severity and hemodynamic management strategies across centers.

Prehospital deaths were not captured in the registry. Consequently, the PRC group was subject to potential survival-to-admission bias because only patients who survived long enough to reach a participating hospital or reperfusion center could be included. Patients with the most severe prehospital shock may therefore have died before enrollment. This left truncation may have resulted in underestimation of the overall mortality associated with shock occurring before arrival and may also have influenced the observed relationship between admission blood pressure and in-hospital mortality.

### Future research

Future research should evaluate the impact of individual BAMT components on long-term outcomes among STEMI-CS survivors in resource-limited settings, particularly after discharge. Given the greater baseline severity observed in the PRC group, longitudinal studies are needed to determine whether the increased use of BAMT for reduced LVEF in this cohort translates into sustained survival benefits. Finally, studies comparing outcomes from this registry with those from centers with interventional capabilities in similar economic settings would be valuable to better quantify the potential benefit of advanced reperfusion infrastructure.

### Conclusions

In this multicenter registry from a resource-limited setting, patients who developed STEMI-CS PRC presented with greater baseline clinical severity, received less inpatient BAMT and streptokinase, and experienced higher unadjusted in-hospital mortality compared with those who developed shock after arrival. However, PRC status was not statistically significantly associated with in-hospital mortality in the multivariable logistic regression model, although the point estimate remained compatible with potentially higher mortality. The observed mortality difference may reflect a combination of shock timing, clinical severity, treatment selection, and residual confounding.

These findings highlight the need to improve early recognition, reduce delays to presentation, and optimize reperfusion pathways for STEMI-CS in settings where pPCI is not universally available.

Perspectives **COMPETENCY IN MEDICAL KNOWLEDGE:** In patients with STEMI complicated by cardiogenic shock in resource-limited settings, shock onset PRC identifies a high-risk subgroup with greater baseline severity, lower use of selected acute therapies, and higher unadjusted mortality.

**TRANSLATIONAL OUTLOOK:** Future studies should evaluate strategies to reduce delays to presentation, improve early shock recognition, optimize fibrinolytic pathways, and assess long-term outcomes after discharge among STEMI-CS survivors in settings where pPCI is not universally available.

**Take-Home Messages**

• Patients who developed STEMI-CS PRC had greater baseline clinical severity than those who developed shock after arrival.
• PRC patients received less inpatient BAMT and streptokinase and had higher unadjusted in-hospital mortality than ARC patients.
• In the multivariable logistic regression model, shock timing was not statistically significantly associated with mortality.
• Improving early recognition, reducing delays to presentation, and optimizing reperfusion pathways may be critical for STEMI-CS care in resource-limited settings.

## Funding support and author disclosures

This research was supported (in whole or in part) by HCA Healthcare and/or an HCA Healthcare affiliated entity. The views expressed in this publication represent those of the author(s) and do not necessarily represent the official views of HCA Healthcare or any of its affiliated entities. The authors have reported that they have no relationships relevant to the contents of this paper to disclose.
